# Supplementary material for: Identification of photoperiod-induced specific miRNAs in the adrenal glands of Sunite sheep (Ovis aries)
Source: Front Vet Sci. 2022 Jul 22;9:888207. doi: 10.3389/fvets.2022.888207 (PMC9354845; doi:10.3389/fvets.2022.888207)
Supplement: Supplementary file 1 [file Data_Sheet_1.ZIP › Supplementary file/miRDeep2 file.zip!/miRDeep2 file/Novel_Structure/SP42A3/Novel_602.pdf]

Provisional ID	: NC_019460.2_6421
Score total	: 1.8
Score for star read(s)	: -1.3
Score for read counts	: 0
Score for mfe	: 1.5
Score for randfold	: 1.6
Score for cons. seed	:
Total read count	: 13
Mature read count	: 13
Loop read count	: 0
Star read count	: 0

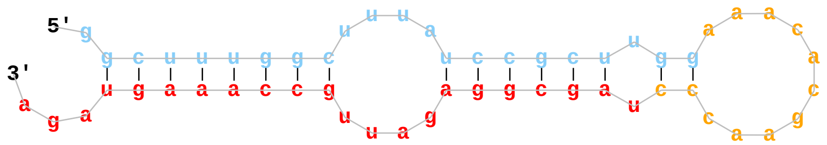

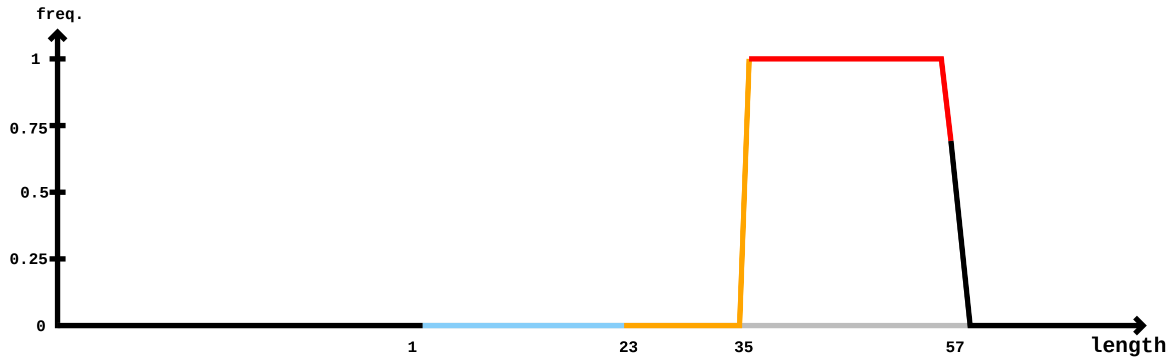

Star Mature

```
5'- gggaaggcaggugaagucuucauucauaaggacaagggcuuuggcuuuauccgcuuggaaacacgaacccuagcggagauugccaaaguagaacuggacaugccacuccgu   -3'    exp
    .(((.((((.((...(((((........)))))....((((((((....((((((.((..........)).))))))....))))))))........)).))))..)))..    reads     mm      sample
    ......................................................................uagcggagauugccaaaguag....................    4         0       seq
    ......................................................................uagcggagauugccaaaguaga...................    9         0       seq
```
